# An improved machine learning protocol for the identification of correct Sequest search results

**DOI:** 10.1186/1471-2105-11-591

**Published:** 2010-12-07

**Authors:** Morten Källberg, Hui Lu

**Affiliations:** 1 Department of Bioengineering, University of Illinois at Chicago, Chicago, IL, USA; 2 Shanghai Institute of Medical Genetics, Shanghai Children's Hospital, Shanghai, PR China; 3 Key Lab of Embryo Molecular Biology, Ministry of Health, China & Shanghai Lab of Embryo and Reproduction Engineering, Shanghai, PR China

## Abstract

**Background:**

Mass spectrometry has become a standard method by which the proteomic profile of cell or tissue samples is characterized. To fully take advantage of tandem mass spectrometry (MS/MS) techniques in large scale protein characterization studies robust and consistent data analysis procedures are crucial. In this work we present a machine learning based protocol for the identification of correct peptide-spectrum matches from Sequest database search results, improving on previously published protocols.

**Results:**

The developed model improves on published machine learning classification procedures by 6% as measured by the area under the ROC curve. Further, we show how the developed model can be presented as an interpretable tree of additive rules, thereby effectively removing the 'black-box' notion often associated with machine learning classifiers, allowing for comparison with expert rule-of-thumb. Finally, a method for extending the developed peptide identification protocol to give probabilistic estimates of the presence of a given protein is proposed and tested.

**Conclusions:**

We demonstrate the construction of a high accuracy classification model for Sequest search results from MS/MS spectra obtained by using the MALDI ionization. The developed model performs well in identifying correct peptide-spectrum matches and is easily extendable to the protein identification problem. The relative ease with which additional experimental parameters can be incorporated into the classification framework, to give additional discriminatory power, allows for future tailoring of the model to take advantage of information from specific instrument set-ups.

## Background

The analysis of composite protein mixtures by use of mass spectrometry techniques has become a standard methodology for characterizing the proteomic profile of a cell or tissue sample [1]. Mass spectral data has proven valuable in addressing complex problems such as the reconstruction of metabolic pathways [2,3] and protein-protein interaction networks [4,5], and is of great utility in applications spanning from the quantification of bacterial proteomes [6] to the investigation of infectious states in soybeans [7].

Efficient use of the MS/MS technique [8] in large scale protein characterization studies requires robust and consistent data analysis procedures. To this end, the combination of spectral data and the vast amount of genomic sequence information available in public databases has proven extremely rewarding. Algorithms such as Sequest [9], Mascot [10], and X!Tandem [11] (amongst others [12,13]) can correlate thousands of mass spectra with theoretically derived peak lists from database peptide sequences, thus effectively automating the interpretation of experimental data. For the above mentioned algorithms, the result of a single spectrum searched against a database typically consists of a set of highly correlated peptide sequences along with a correlation score and a number of additional metrics intended for validation of the specific peptide-spectrum match.

There is, however, often no direct interpretation of these scores in terms of statistical significance [14], therefore simply ranking well-correlated peptides by metrics provided from the initial database search procedures and selecting a cut-off for filtering true matches from false ones is not desirable. Depending on the choice of threshold such a procedure will either be too conservative or yield a high rate of false-positives [15]. On the other hand, manual validation of the large amount of data produced by MudPIT style [16] experiments would be time consuming and out of tune with the high-throughput experimental work-flow characterizing the field at present. Thus, to ensure an effective production pipeline, a fully automated method for confident validation of the results produced by the above mentioned search algorithms is essential.

A number of procedures for validating peptide-spectrum matches have been suggested, either as direct extensions of the Sequest or Mascot algorithms or as supplementary post-processing tools [17-20]. Our focus here will be on the analysis of the search results produced by the Sequest algorithm [9], and how to efficiently improve the number of true peptide-spectrum matches identified at a controlled false positive rate.

Currently, the most widely used tool for evaluating Sequest search results is the PeptideProphet methodology developed by Keller *et al*. [21,22]. By use of an empirically determined probabilistic mixture model based on the fitting of assumed distributions of various metrics (believed to reflect the reliability of the spectrum-peptide match) the search results are evaluated. The procedure returns a probability estimate of a peptide being present given the database search results. While giving much higher sensitivity measures than simple threshold based methods, this approach does suffer from two short-comings: First, there is no theoretical work supporting the assumptions made regarding the distributions used to fit the features utilized. Second, the model may not be easily extendable when potentially discriminatory information from novel types of data become available.

Machine learning provides an attractive platform for addressing the above concerns since no prior assumptions about the distribution of the individual features have to be made. In addition, the flexibility in feature handling of most machine learning algorithms makes further improvement of predictive power and robustness straight forward as new information becomes available. In recent years a number of bioinformatics problems have been addressed using machine learning [23], for example, the prediction of protein-DNA interactions [24-26] and protein-membrane interactions [27]. Likewise, previous works have used machine learning methods for identifying true peptide-spectrum matches through different formulations of the problem. Anderson *et al*. [28] were the first to apply such procedures to mass spectral data in their study of Support Vector Machines (SVM) classification of Sequest search results from iontrap data. Razumovskaya *et al*. conducted a similar study demonstrating how a neural network could improve the filtering of Sequest search results over simple threshold-based procedures. In a study using ion-trap data Elias *et al*. demonstrate how the identification of peptide-spectrum matches can be improved through probabilistic modeling of fragment intensities observed in the spectrum at hand [29]. Ulintz *et al*. [30] developed an approach using tree-based ensemble algorithms and demonstrated that these were superior to the SVM protocol used in previous studies. A recent study have further demonstrated how physiochemical properties of the peptide in question can provide discriminatory power between true and false matches without using database search engine scores [31].

From the above review it is clear that a variety of supervised classification regimens, using many different sources of information, have been tested thus far. Here we present a work that improve on three separate aspects on the above mentioned machine learning procedures for identifying correct peptide-spectrum matches from the Sequest database search procedure.

First, our classifier performs 6% better as measured by the area under the ROC compared with results by Ulintz *et al*. [30] on the same dataset. The improvement is achieved by introducing a number of global dataset features that take into consideration factors such as the total number of peptide-spectrum matches belonging to a given protein and the percentage of potentially observable peptide sequence from a given protein actually appearing in the search result.

Second, by using the Alternating Decision Tree (ADTree) [32] classification algorithm we are able to represent the developed model as a tree with a limited number of nodes, thereby rendering the model interpretable to humans. While this trade does not add anything in terms of predictive power, interpretability of the model makes the procedure clearer to experimentalists and allows us to compare the prediction rules to expert rule-of-thumb, giving an empirical validation of such rules.

Third, we build a straight-forward probabilistic procedure for extending the machine learning identification of the peptide-spectrum matches into the protein prediction problem (i.e. identifying the proteins contained in the initial sample), by converting the classification scores into true probability estimates by means of logistic calibration. The latter of the two problems is often of most interest to experimentalists, as one is interested in knowing the probability of a protein being in the sample, not simply which peptide fragments were confidently identified.

## Methods

### Reference Dataset

Our method was tested using a publicly available MALDI MS/MS dataset obtained from a sample of 246 known proteins [33] published on ProteomeCommons.org [34]. The peaklists were searched using the Sequest algorithm [9] on the IPI human FASTA database ver. 3.14 (for comparison with results reported by Ulintz *et. al *[30]) with the post-translational modification methylation, oxidation, and phosphorylation. Comparison with the PeptideProphet [21,22] validation results of the Sequest output was done using output from ver. 4.0. The PeptideProphet ROC curve and evaluation metrics reported below were obtained from this output.

To correctly evaluate our approach the original dataset was split into two, one for method validation and one for training the machine learning protocols. Each of these datasets consists of a total 43,348 examples of which 2,035 are correct peptide-spectrum matches. In contrast to the works we are comparing with [30] all 10 top-ranked tentative peptide matches from each spectrum searched are included in the training and testing set. Including all potential matches is important, as 34% of the true matches have been found not ranked first. Furthermore, only including the top one or top five ranked matches will exclude some potentially difficult instances that may add valuable information for identifying novel proteins.

### Classification Algorithms

Models were constructed using four different binary classification procedures, namely AdaBoost [35] applied to C4.5 [36] and Willow tree [37], Random Forest [38] applied to C4.5, and Alternating Decision tree [32] (in the following denoted ABC4.5, ABWillow, RFC4.5, and ADtree, respectively). All algorithms used in this study are supervised classifier, a model does thus need to be trained on a labeled training dataset (training mode) and can thereafter be used to predict new examples without further parameter tuning (prediction mode). Casting the problem in a binary classification framework, we refer to each peptide-spectrum match as an instance (in the dataset), with the *i^th ^*instance consisting of a feature vector *x_i _*∈ [1 × *n*] and a label *y_i _*∈ {0, 1}, with *n *denoting the feature count. All algorithms described construct a function, *g*(*x*), that minimizes the empirical risk of misclassifying an instance, under the assumption that all instances are drawn with respect to the same (unknown) probability distribution. In the following we limit ourselves to describing conceptual details of the utilized algorithms, referring the reader to cited works for technical details.

C4.5 and Willow tree are both decision trees algorithms iteratively growing a classifier tree by finding splits of the dataset with respect to the feature value which results in the greatest gain in Shannon entropy (a function used to quantify how homogeneous the instances reaching a certain leaf node in a tree classifier are with respect to instance label). The procedure halts when all instances in a leaf node are of the same class or a pre-defined stopping criterion has been reached.

We apply two so-called meta-classifier techniques to the above mentioned tree algorithms, namely AdaBoost [35] and random forest [38]. Both work by training a collection of decision trees over iteratively modified versions of the original training set and combining the prediction power of these models into one superior ensemble-classifier. The AdaBoost procedure iteratively updates importance weights for the dataset instances for each tree model constructed during training. The distribution of weights is changed such that higher weight is given to instances misclassified in the previous iteration. The final classification of an instance is made by the majority vote on classes returned by the tree collection. In the case of Random Forest each tree is trained on a bootstrap sample of the available instance and each node split only considers a number *m *of the available features (where *m << n*). The final class label of an instance is assigned by taking the mode of the class labels returned by the constructed tree set.

**Figure 1:**
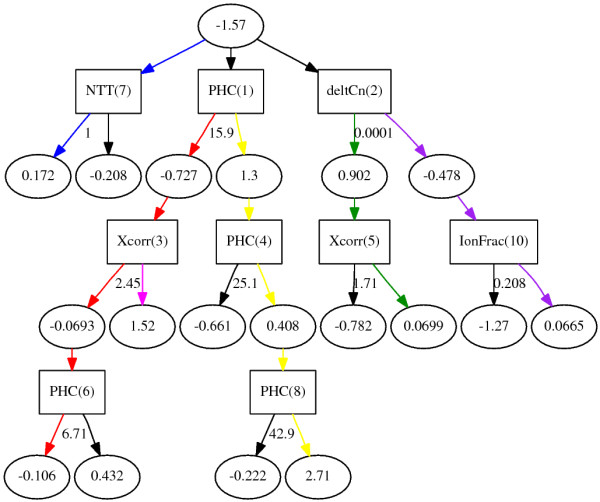
**Graphical representation of the alternating decision tree learned from**. Prediction nodes are represented by ellipses and splitter nodes by rectangles. Each splitter node is associated with a real valued number indicating the rule condition, meaning: If the feature represented by the node is less than or equal to the condition value the prediction path will go through the left child node, otherwise the path will go through the right child node. The numbers behind the feature names in the prediction nodes indicate the order in which the different base rules were discovered, this ordering can to some extend indicate the relative importance of the base rules. A detailed explanation on how to interpret the ADTree is given in the main text along with a discussion of the colored paths outlined. tree.png.

The ADtree algorithm also utilized the AdaBoost technique, but unlike ABC4.5 and ABWillow it has the advantage of producing models that are easily represented as a tree with a limited number of nodes (less than 20). This property is achieved by constructing a tree that is a conjunction of rules which all contribute real-valued evidence toward a given instance being classified as either true or false. Unlike traditional tree models the classification of instances by ADtree is thus not determined by a single path traversed in the tree, but rather by the additive score of a collection of paths. The ADtree is graphically represented with two types of nodes: Elliptical *prediction nodes *and rectangular *splitter nodes *(see Figure 1 for an example). Each splitter node is associated with a value indicating the rule condition: If the feature represented by the node is less than or equal to the condition value for a given instance, the prediction path will go through the left child node, otherwise the path will go through the right child node. The final classification score produced by the tree is found by summing the values from all the prediction nodes reached by the instance, with the root node being the precondition of the classifier. If the summed score is greater than zero, the instance is classified as true.

In addition to providing a classification label, the tree score of an instance (the margin score) is a measure of confidence in the classification label, a feature that makes is possible to convert these into true probability estimates. To this end we use Logistic calibration [39], providing a one-to-one mapping between the marginal score and a probability estimate.

### Evaluation Metrics

All instances will be classified into one of the following categories: True Positive (TP), False Positive (FP), True Negative (TN), or False Negative (FN). By determining the count of instances in each category, the following quality metrics can be estimated:

$$\begin{array}{c} Accuracy=\frac{TP+TN}{TP+TN+FP+FN}\\ Sensitivity=\frac{TP}{TP+FN}\\ Specificity=\frac{TN}{TN+FP}\\ Net.\;prediction=\frac{Sensitivity+Specificity}{2} \end{array}$$

Additionally, the area under the curve (AUC) of the receiving operator characteristic (ROC) is used to have a metric that is unbiased towards the class distribution of the dataset. The ROC is defined as the (1-specificity, sensitivity)-curve, with each point corresponding to a specific threshold for class separation. An AUC value of 1 corresponds to an error-free performance over the entire range of thresholds, whereas a random classifier achieves an AUC value of 0.5. In addition to the AUC measure we use Precession-Recall Curve (PRC) ($\text{precision}=\frac{TP+TN}{TP+FP}$ and recall = sensitivity) to judge whether a classifier is truly superior to another, as it has been shown that domination in ROC-space does not always result superiority in PRC-space [40].

### Availability and requirements

The MALDI TOFTOF dataset used for constructing and validating the procedure is publicly available on ProteomeCommons.org https://proteomecommons.org/dataset.jsp?i=71683[33,34]. The code for constructing the models presented is freely available as part of our in-house machine learning workbench, MALIBU [37], available at http://proteomics.bioengr.uic.edu/malibu/. MALIBU is used for both training and validation of the classifiers. All algorithm parameter tuning was done with standard settings for the MALIBU package [37].

## Results and Discussion

The developed machine learning protocol for identification of true peptide-spectrum matches was constructed in three steps: calculation of features for representation of each instance in the dataset; construction of classification models based on the annotated instances; and evaluation and interpretation of the resulting models. In the following, we present the details of each step and describe a method for extending the developed protocol into a probabilistic protein identification method.

### Feature Calculation

A summary of the features utilized in this work can be seen in Table 1. We divide the features into three groups reflecting how they are derived. The *Sequest *group contains features that can be obtained from the output of the Sequest algorithm, such as the correlation score (*XCorr*) between the theoretically calculated and experimentally obtained spectra and the difference between parent ion mass and database peptide mass, (*deltaMH*) amongst others (*Sp*, *SpRank*, *deltaCn*, *ionfrac*). As these values are well characterized elsewhere [9,21] we will not go into further detail here.

**Table 1:** Features used in the machine learning formulation

Group	Name	Meaning	Origin
SEQUEST	XCorr	Rank score from the SEQUEST search.	SEQUEST
	deltaMH	Difference between mass of parent ion and identified peptide mass.	SEQUEST
	deltCn	Difference between XCorr of the highest ranked peptide and the peptide in question	SEQUEST
	SP score	Preliminary score of peptide in search procedure	SEQUEST
	SP rank	Initial rank of peptide based on SP-score	SEQUEST
	Ion fraction	Percentage of ions in the mass spectra that could be correlated with the spectrum	SEQUEST

Published	Number of tryptic	Number of tryptic cleavage sites in the peptide targets (NTT)	Calculated
	Peptide lenght	Residue count of the peptide	Calculated
	Summed Intesity	Sum of peak intensities in the spectra	Calculated
	Mobil proton factor (MPF)	Measure of the proton mobility in peptide	Calculated
	C-terminal Residue	Amino acid residue at c-terminal (Arg = 1, Lys = 2, Other = 3)	Calculated
	Mass-window peptides	# of DB peptides within prespecified mass-window of the parent ion	Calculated
	Proline count	# of Pro residues in the peptide	Calculated
	Arginine count	# of Arg residues in the peptide	Calculated

Novel	Intensity Mean	The mean of the peak intensities	Calculated
	Intensity Std.	Std. of the peak intensities	Calculated
	Intensity bins	The distribution of intensities in 20%-bins	Calculated
	Protein Hit Count (PHC)	Probability score of observing × number of peptides from parent protein	Calculated
	Potential Coverage Ratio	The potential sequence coverage	Calculated
	PTM percentage	The percentage of possible PTMs found in a peptide	Calculated

For each individual feature we give a brief description and indicate whether the feature was obtained from the output of the SEQUEST algorithm or calculated from the identified peptide, the mass spectrum, or database statistics. The features have been divided up into three subgroups *SEQUEST*, *Published*, and *Novel*, denoting those features that can be derived directly from the SEQUEST algorithm output, those used in published studies of the identification problem, and those introduced in this work, respectively.

The *Published *group contains features that have been used in previously published results on classifier construction for the problem at hand. The computations needed to derive these features are self-explanatory given the description in Table 1. We will refer the reader to the study by Ulintz *et. al*. for further details on computation and the underlying intuition leading to the inclusion of these features [30]. The *Novel *group consists of features not previously included in other machine learning formulation of this classification problem, and includes features for quality assessment of the spectral data as well as probability measures specifying the likelihood of observing the entire dataset. Six novel features are calculated and their rationale is described below.

Intuitively, one would be more confident in identifying a borderline peptide-spectrum match as being true if other peptides from the same parent protein are observed in the search result. In other words, given prior knowledge, one would favor specific peptide-spectrum matches over others with similar correlation values, due to our overall knowledge of the search result. This intuition leads to the implementation of two novel features, namely the *Protein Hit count *(PHC) and the *Potential Coverage Ratio *(PCR).

We formulate the PHC as the following probability: Given a database containing a certain number of observable peptide *D *(with respect to the mass limitations of the instrument used for analysis, the digestion enzyme utilized, and the post-translational modifications specified in the database search) and a search result containing *P *samples from this database, we want to calculate the probability that *k *or fewer observations of a given protein would be made by randomly sampling from this database. For each peptide stemming from a protein that has been matched *k *times in a search we will specify the PHC by the binomial distribution, where *n *is the number of potentially observable peptides from this protein:

$$PHC=\sum_{i=1}^{k}\left(\begin{array}{c} \text{D}\\ \text{P} \end{array}\right)\;{\left(\frac{n}{D}\right)}^{i}{\left(1-\frac{n}{D}\right)}^{P-i}$$

The above probability is estimated using a Poisson distribution and is reported in negative log-space in order to avoid numerical artifacts. Notice that since both the database size and the number of spectra are included in the calculation of the above term, any learning algorithm trained on a specific training set with given a database and a collection of spectra should work equally well on datasets obtained from a different database size searched with a different number of spectra.

One concern that may be raised when utilizing information from the parent protein, as is the case with PHC, rather than the peptide-spectrum match itself, is how such features will handle the fact that some peptides can be mapped to several parent proteins due to the existence of orthologs and homologs in the database searched. One should, however, recall that a spectrum and a peptide fragment match provided by the Sequest protocol is always linkable to the specific parent protein that gave rise to the theoretical peptide-fragment matched to the spectra. Consequently there is never any doubt which parent protein the specific peptide-fragment should be counted towards. In fact, in instances where two proteins (one present in the sample and one not present) have a certain degree of sequence similarity the PHC may actually help weed out false-positive hits from distant homologs as hits from such homologs will have a lower PHC (and PCR) than hits from the protein actually present in the sample.

The PCR is simply defined as the percentage of residues belonging to observable peptide fragments that are observed in the set of peptide-spectrum matches from the Sequest search. Further, we include the PTM percentage, which denotes the percentage of potential post-translational modifications (given the current search settings) included by Sequest to obtain the present correlation scores. The logic behind including the PTM percentage is as follows: PTMs are often functional modifiers of proteins. The need for the hypothetical inclusion of a high-percentage of the potential PTMs in a short peptide fragment in order to get a good correlation with the spectra at hand could indicate that the match is not a true-positive as it seems unlikely to have a large number of functional modifiers close together in a relatively short peptide-fragment.

Previous works [41,42] have shown that an automated quality assessment of the spectral data can help validate peptide-spectrum matches by sorting out low quality spectra. The simplest features incorporating this notion are *Intensity mean *and *Peak count*, which specify the average intensity of all peaks in the raw spectrum and the total number of peaks, respectively. Both of these values are often used in human assessment of spectral quality [43] and have discriminatory power in sorting out spectra of poor quality [42].

### Classifier Performance

We compare the performance of a collection of classification algorithms using datasets including different subsets of features. One set includes the *Sequest *and *Published *feature-groups from Table 1 and another one includes all features, referred hereinafter as the *S+P *dataset and *All *dataset, respectively. Each dataset is divided into a training set for classifier construction and parameter tuning (by means of cross-validation), and a distinct test dataset for evaluating the classifier performance. We choose to evaluate our method using a test set rather than by using cross-validation on the training set to ensure that dependencies between features from different instances within the dataset do not inflate the performance metrics (this concern is particularly relevant for the PHC feature).

**Figure 2:**
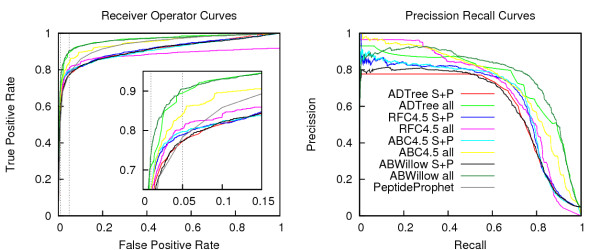
**Receiver Operator Curves (ROC) (left) and Precision/Recall Curves (PRC) (right)**. Classifiers trained with the novel set of features have the suffix *all*, otherwise the suffix *S+P *is used (this does not apply to the curve for PeptideProphet shown in the ROC plot). The ROC shows how the TPR varies with the FPR, indicating what percentage of true hits one can expect to obtain at a given false-positive-rate. The PRC given an alternate view of the classification depicting the precision as a function of the recall (note PeptideProphet results only shown in ROC). rocspr.png.

Table 2 shows the performance of a number of classifiers on the *S+P *and *All *datasets. The high ratio between negative and positive instances in the datasets means that accuracies correlate strongly with prediction performance on negative cases. Consequently, the accuracy and specificity metrics, which for both datasets are well above 98%, are not instructive for comparing the performance. Better comparison can be made with Net Prediction and AUC, as they are insensitive to skews in class distribution. Gauging these metrics, it is clear that the novel features introduced in this work provide added discriminatory power between true and false instances. The best performance is achieved by the ADtree algorithm with the *All *dataset, giving a 6% higher AUC ROC than the best performing algorithm in on *S+P *dataset. When comparing the performance of the same algorithm on the two dataset, we observe that 3 out of 4 algorithms perform better on the *All *than on the *S+P *dataset, a fact that is also clearly illustrated in the ROC curves in Figure 2 (left). Here we observe that the ADtree and ABwillow algorithms applied to *All *dataset outperform all other classifiers over the entire range of False Positive Rates (FPR), whereas the ABC4.5 on the *All *dataset falls somewhere in between these two and the results from classifiers trained on the *S+P *dataset. In addition, all classifiers trained on the *All *dataset perform better than the PeptideProphet procedure over the entire FPR range. When comparing the classifiers trained on the *S+P *feature collection to the PeptideProphet result, the picture is not as clear. As can be seen on the enlargement in Figure 2 (left), the machine learning algorithms do in general (regardless of feature set) perform better than PeptideProphet at lower FPRs, while PeptideProphet gives better sensitivity at higher FPRs (Note, the high FPR range is rarely used in real applications). We also note that the results obtained on the *S+P *dataset containing the same features as utilized by Ulintz *et al*. closely match the result reported on a preliminary version of mass spectral data used in this study [30]. The PRC depicted in Figure 2 (right) offers an alternative view of classifier performance. The plot does not allow for judgment of which algorithm does better on a specific dataset, as all show strengths and weaknesses at different recall values. It is, however, clear that all algorithms trained on the *All *dataset do better than the ones trained on the *S+P *dataset, conforming the discriminatory power of the new features introduced in this work.

**Table 2:** Validation metrics for a collection of machine learning algorithm runs over testsets containing feature from the groups denoted in table 1

Feature groups	Algorithm	Accuracy	Sensitivity	Specificity	AUC ROC	**Net pred**.
*All*	ABWillow	0.97505	0.56504	**0.99385**	0.96379	0.77945
	ABC4.5	0.97361	0.58815	0.99269	0.94821	0.79042
	RFC4.5	0.97276	0.57212	0.99259	0.87901	0.78235
	ADtree	**0.97688**	**0.7248**	0.98988	**0.96923**	**0.86118**

*SEQUEST*	ABWillow	0.96951	0.48762	0.99336	0.90723	0.74050
	ABC4.5	**0.97258**	0.57018	0.99250	0.907084	0.78139
*Published*	RFC4.5	0.97228	**0.58961**	0.99122	**0.912744**	**0.79042**
	ADtree	0.96925	0.48762	**0.99310**	0.90604	0.74032

-	PeptideProphet	0.9688	0.54	0.99	-	0.765

### An Interpretable Model

As observed above, the ADTree algorithm is among the strongest performers on the dataset incorporating all features, rivaled only by ABWillow tree. In comparison with machine learning algorithms such as SVM, the ADTree algorithm provides the advantage of being represented as a collection of user interpretable rules. Figure 1 shows a graphical representation of the ADTree model learned from the *All *dataset (see **Methods **for how to interpret the tree).

The base-rules in the tree are numbered in accordance with their order of discovery (the number indicated in parenthesis after each feature name), which can be interpreted as the rule importance or predictive power of the feature [32]. Given that interpretation, surprisingly, the PHC appears to have the strongest discriminatory power amongst true and false instances in that a *PHC >*15.9 adds significant weight towards a positive prediction (the final faith of an instance satisfying this rule is of course also based on the other base rules involving PHC). Thus the learned model suggests that a higher than expected number of peptides from one protein in the Sequest search result, is indicative of these peptide-spectrum matches being true hits. The second and third base rules discovered are cut-offs for the *XCorr *score and *deltaCn*, two of the main attributes of the Sequest algorithm used for judging how well the theoretical peptide spectrum correlates with the experimentally obtained spectrum. (It should, of course, be noted that except for rules with the root node as parent, the prediction bias of a rule should always be seen in context of its parent node(s)).

To better interpret the possible paths traversed by a dataset instance, subsets of base-rules have been highlighted in color in Figure 1. We will now examine these paths more closely to see how the classifier is able to discover meaningful knowledge, while at the same time providing high accuracy classification results.

The blue path is made up of only one feature, namely NTT. If the peptide has at most one missed cleavage side, this provides evidence toward the hit being positive, though an instance satisfying this requirement could still ultimately be classified as a false hit. If we examine the red path we see that a *PHC <*15.9 is negative evidence towards the hit being true, as it is unlikely that we would observe only a few peptides from a protein that is indeed present in the sample. If the peptide-spectrum match does, however, have a strong correlation score (*XCorr >*2.45) this effect is reversed, giving the path a net positive score. Interestingly, an *XCorr *score lower than the 2.45 threshold does not add significant evidence toward the match being false. Thus high *XCorr *scores add evidence towards an instance being true, while scores below constitute a borderline region where other factors determine the faith of the instance.

The fact that the PHC feature only comes into play when the *XCorr *score is below as certain threshold is an important model feature, as the PHC score might otherwise "hurt" the classification of proteins with few "mass specable" peptide-fragments. The PHC is, in other words, not used unless the quality metrics correlating the spectrum and the proposed peptide do not provide sufficient evidence to conclusively determine whether the peptide-spectrum match is correct. In situations where there are only one or two "mass specable" peptides from a protein one would want the quality metrics of matches to be highly confident when using them to identify the parent protein, the strategy learned by the model is thus reasonable when handling such instances.

A related mechanism is observed when following the green path, here *deltaCn *values of at least 0.05 add evidence toward the instance being a true hit. The following *XCorr *filter shows that correlation values below 1.71 are strong evidence towards the instance being negative, values above this threshold do not add evidence towards the instance being positive. The yellow path does not add any new features to the classifier, but simply acts as a further filter on the *PHC *feature, constructing intervals with increasing summed evidence towards the instance being positive. The purple path, on the other hand, adds two new features. If the instances following this path has a *deltaCn <*0.05, and at the same time an *IonFrac *value of less than 20.8%, there is substantial evidence towards the instance being false, whereas higher IonFrac values are indicative of a true instance when combined with a low *deltaCn*. In other words, small differences in the mass of parent ion of the mass spectrum and the theoretical mass of the peptide that it has been matched with is a strong indicator of a true hit only if a certain fraction of the spectral peaks are accounted for by that specific peptide.

We observe that none of the features intended to address the issue of spectral data quality were found to be instrumental in significantly improving the classification accuracy for the ADtree model. This is somewhat surprising, since well above 85% of spectra were considered to be of bad quality in studies addressing the problem of identifying such cases [41]. Thus, one would expect that a feature identifying such spectra would provide certain discriminatory power. One possible explanation for this observation is that these cases are already covered by other rules from the ADtree, thus including the spectral quality feature to the model would not add additional predictive power. For instance, one might reason that cases with inferior spectral quality will only give rise to database hits with low *XCorr *score, which would render these cases false hits due to this feature.

The rules discovered above using the ADTree agree well with expert criteria previously used as conservative estimates for identifying hits that would be true with high probability. Washburn *et al*. [16] did, for instance, settle on the following conjunction of rules as criteria for correct hits: *XCorr >*2.2, *deltaCn <*0.1 and the peptide has to be fully tryptic (meaning *NTT *= 0). The classifier developed is comprised of rules with similar cut-off values for the features used by experts, but does also utilize novel rules when making predictions, identifying true instances that would otherwise have been missed. Take for instance the *XCorr *cut-off: We found that values above 2.45 provide strong evidence towards an instance being a correct match. If the value, on the other hand, is below this cut-off we did not find it to be significant evidence toward the hit not being correct unless the value fell below 1.71, providing room for a number of borderline instances that can be correctly classified using the additional features in the model.

### Extending the Peptide Prediction Protocol to the Protein Prediction Problem

The ultimate goal of MS/MS experiments is not necessarily the confident identification of peptides, but rather determining a probability measure for the presence of their parent proteins in the sample analyzed. One software application addressing this issue is the ProteinProphet software [44] by Nesvizhkii *et al*., which identifies a minimal set of proteins accounting for the observed peptides by use of the expectation-maximization algorithm. Following the formulation in this work we show a straight-forward way of extending our peptide identification protocol to a protein identification protocol.

A conservative estimate of the probability, *P*, that a given protein is present (meaning that at least one of the peptide matched by the database search from this protein is correct) is given by

(1) $$P=1-\prod_{i}(1-\underset{j}{\max}p(+|D_{i}^{j}))$$

where the product index *i *is over all distinct peptides from this protein, the index *j *is over all matches obtained for one specific peptide and $p(+|D_{i}^{j})$ denotes the probability of the *j^th ^*identification of peptide *i *being a true match. We take the maximum over all identification from all identical peptides, as these should not be considered independent. Note further that this formulation theoretically allows for a specific peptide to be considered as evidence for two distinct proteins.

By use of logistic calibration we convert the classification scores obtained from the ADtree algorithm into probability estimates of a given peptide-spectrum match being correct (or in other words we estimate $p(+|D_{i}^{j})$). Combining these estimates with (1) we can calculate probability estimates for each protein that has at least one peptide identified in the database search actually being present. Using this relatively simple extension of the classification framework we are able to identify 87% of the proteins present in the sample at false positive rate 5%. In comparison, using the probability estimates from PeptideProphet only achieved an identification rate of 85%. This is not surprising as we previously saw that the ADtree procedure identifies more correct peptide-spectrum matches than PeptideProphet.

## Conclusions

Supervised machine learning provides an attractive platform for examining the peptide prediction problem since no prior assumptions of the distribution of the utilized features have to be made when constructing the model. This is in contrast to generative/unsupervised models such as the PeptideProphet procedure, that assumes specific distributions (in this case Gaussian and Gama distributions) when classifying matches. While it has been shown that the assumption regarding a specific data distribution is reasonable [21,44] in certain instances of the identification problem there is no general evidence or theoretical framework supporting this claim for all types of instrument or data. As conveyed in this work another attractive property of the supervised machine learning framework is the relative ease with which the developed models can be extended with novel features in order to improve predictive power. It thusly becomes possible to construct a tailored peptide identification framework for specific experimental procedures and equipment choices, thereby providing stronger guarantees on the control of error rates than would be possible with a generic setup. One drawback of the supervised learning approach is of course the need to construct a training dataset from a know protein sample to do the initial parameter tuning of the model and determine the performance metrics. However, once the training is done the trained model will perform equally well on large scale and sparse datasets, since one does not have to be concerned with having too little data to properly estimate model parameters.

Since large-scale proteomics studies are often concerned with characterizing the proteomic make-up of the cell in a number of states, a reliable probabilistic measure for the presence of a given protein is essential. Above we demonstrated how predictions from the ADtree model (or any other supervised learning algorithm providing marginal classification scores) combined with logistic regression can be used in a simple probabilistic framework to give a high protein identification rate at a low FPR.

In sum, we have improved on previously published machine learning procedures for identification of correct peptide-spectrum matches by introducing novel features adding to the predictive power of all the tested algorithms. Furthermore, we have introduced the ADtree procedure into the problem domain, constructing an interpretable model that correlates well with previously published rules addressing the classification problem at hand. Finally, we show how the protein prediction problem can be addressed within the presented framework.

In this work we demonstrate how a generic classification model for MS/MS data obtained by use of the MALDI ionization can be constructed. In future work, we intend to extend the classification framework to take advantage of experiment specific parameters (ionization method, instrument type, pre-processing steps of the sample) creating models tailored specifically to the instrumental set-up used to obtain the spectral data.

## Authors' contributions

MK conceived the idea, designed the study, carried out the analysis, and drafted the manuscript. HL conceived of the study, participated in its design and coordination, and helped draft the manuscript. Both authors read and approved the final manuscript.
